# Peru and its new challenge in higher education: Towards a research university

**DOI:** 10.1371/journal.pone.0182631

**Published:** 2017-08-07

**Authors:** Carlos Lavalle, Victor Luis de Nicolas

**Affiliations:** 1 Departamento de Ciencias Económicas, Universidad de Piura, Lima, Perú; 2 Grupo Gesplan-UPM, Universidad Politecnica de Madrid, Madrid, España; International Nutrition Inc, UNITED STATES

## Abstract

The paradigm of research universities linked to the emergence of university rankings has unified and universalized the criteria relating to the quality of higher education. This situation has led to multiple responses across global society, which has started rating the quality of higher education systems through these rankings, supported by a series of indicators aligned to the characteristics of research universities. Given that the quality of a country’s higher education is one of the fundamental pillars of its development, many countries have started to take government action in this respect. In the case of Peru this has not taken long, with the approval of Law 30220 in 2014. This aims to regulate the quality of higher education through a series of specific conditions governed by a newly created body known as SUNEDU. This article uses a Delphi panel to analyze the existing relationship between the conditions imposed by SUNEDU and the research universities’ intrinsic characteristics. During the Delphi panel a consensus was reached through an acceptable and stable level of responses, resulting in confirmation that there is alignment between the conditions imposed by SUNEDU and the intrinsic characteristics of research universities.

## Introduction

In this century, the scope of universities is no longer seen as merely preserving knowledge, nor as educating the elites in order to strengthen nations. Instead, they are seen as incubators of strategic resources, innovations and new technologies which, as business projects, provide results that extend further than a specific geographic area [1–3]. In the modern day, this model has been adopted by the so-called “world-class universities”.

During the first decade of the 21st century, and as a result of the emergence of international rankings, the term “world-class university” became a fashionable term [4]. Since then, a number of studies emerged which tried to analyze the characteristics and unique aspects of these world-class universities. According to several comparative studies from the World Bank, many of the attributes of traditional universities with a global standing, classified based on the rankings, can be applied to research universities. One of the best known authors [5] states in his reports that natural evolution during this era has complemented the traditional focus of universities (teaching) and driven this more towards research. Institutions that focused on this now find themselves in dominant positions compared to others [6]. On this point, the expert clarifies that research can only lead to a university being recognized if it is “a study which breaks down knowledge barriers, which can be measured and communicated” [5]. It is as a result of these studies that the term “world-class university” starts to be aligned with the concept of research, which in this last decade has led to a new international paradigm derived from the North American research intensive university concept: The research university.

The emergence of these international rankings in 2003 has catapulted research universities as a benchmark for university quality. The high praise which these university rankings give to research has led to the universities which appear in the top spots of these rankings being universities in which research plays a major part in their operations [6–7]. As a result, research universities have become the new world-class universities, with the perception of university quality focusing on this new aforementioned paradigm: the research university [8]. This has led to a large range of opinions amongst the scientific community, which has created a debate in terms of what these universities contribute in the context of the country [9].

In many developed countries, there is a consensus that research universities have contributed to a modernization of disciplines and specializations in academic and scientific fields, whilst at the same time strengthening knowledge in new areas and understanding more complex situations. There is also a new point of view that proposes that the knowledge developed in the 21st century seems to come from the limitations of these same studies and advises the convenience of demanding other new ones to complement and strengthen them [4]. This process has led research universities towards a new paradigm from which certain questions arise, such as whether their existence as an institution is linked to the needs of the communities which they serve, and whether they promote a type of education which guarantees equal opportunities for everyone [8].

Based on this, it is understood that establishing a research university in a country does not just depend on there being recognized talent amongst the population, political will or the necessary resources to build such a university. In reality, the most complex aspect of creating a world-class university in a country is the creation of a long-term vision [10]. As an example, this plan is required in order to develop a model which is capable of enabling an institution to be aligned with the social development and economic strategy adopted by the country. Other necessary considerations include reviewing their interaction between the country’s education system, which should also already be in a phase of reassessment and reform. This is required in order to guarantee the efficient running of a world-class university and links with other institutions. The objective is to create effective feedback in terms of learning, research and technology transfer, and achieve a positive impact within the local and regional environment. If there has previously been no focus on all of these key factors mentioned, simply creating a World-Class or Research University could facilitate, but does not necessarily guarantee, a country’s development and productivity [4][11].

As a result, the upcoming years will be crucial in different regions such as Latin America, where, since the 19^th^ century, universities have been adopting a university model referred to by some authors as Napoleonic [11–12]. This model has more of a pragmatic focus, rather than critical, and is focused on professional training and issuing degrees in which the teaching aspect has prevailed. There is doubt over its quality compared to research and creating new knowledge [4][13]. Therefore, many of these countries (including Peru) have implemented a series of university reforms which starts a process aimed at reaching universities. Not only does this ensure a good standard of learning, it also incorporates and strengthens the concept of research. This research should be seen as a crucial element in the country’s development, as well as their own [14] as it has the ability to act as a communications channel between these countries and universal science [15]. This article analyzes the focus of Peru’s university reforms, evaluating the existing link between conditions imposed by SUNEDU (Superintendencia Nacional de Educación Superior Universitaria) through Law 30220 (as quality indicators for higher education) and the characteristics of the research universities which occupy the top spots in the international rankings.

## Characteristics of a research university

The World Bank reports are conclusive: thinking about research universities is currently equivalent to thinking about creating world-class universities. The high level of qualifications and achievement possibilities which graduates from these organizations benefit from, the prestige associated with the publications presented by students and teachers on their research and studies, and the effectiveness and transferability of knowledge which they provide both the public and private sectors, demonstrate that research universities are a new educational model which takes knowledge and makes it interact at different levels within the global market [4] [16–17]

Based on this focus, the operations of research universities depend on the following factors: talent potential amongst teachers, researchers, students and managers of the funds with which the institution is run, successful and flexible governance which enables them to reestablish themselves across different cultural and political contexts without sacrificing their academic and financial autonomy as well as their organizational vision, and abundant resources (in the form of donations, contributions and investments from the public and private sectors) which enable the deployment of tools and spaces which facilitate research and experiments [4] [18–19].

Following a project with various universities (University California Los Angeles, University California Berkeley, Universidad Politécnica de Madrid, Universidad Politécnica Salesiana & University California San Diego), [20] work which was published in 2014 emphasized the gradualness of the concept of research universities as an objective which should always be considered with a strategic vision. As an example, research universities have a greater focus on postgraduate studies rather than degrees, but that does not mean that they only provide postgraduate studies. As a result, a university which wishes to change can start with an international level postgraduate degree which can gradually be strengthened and improved. This report, [20], proposes three key characteristics which research universities should have: relevant teaching, research and links with society. These three characteristics should not be looked at in isolation, as there are links between them; grouping, broadening and rejecting aspects proposed by different researchers [4] [19]. Therefore, these characteristics build on the aforementioned factors, as they form objectives and action models which are capable of providing guidance and clarifying what exactly a research university is in reality.

### Relevant teaching

Teaching has been, is, and will be one of the main components of the university concept. The university is intrinsically linked to teaching as a pillar for learning and transferring knowledge. The fact that this teaching is relevant contributes to the strengthening and prestige of the institution in which it takes place.

When it comes to considering which teaching is relevant we should turn to the concept of the university itself, which refers to the universality of the entity. Relevant teaching is not isolated, it is international (universal) and is not an end in itself but rather a way of transmitting existing knowledge, not only for teaching but also for teaching how to reflect, in order to develop. This development objective requires the teaching to be current, suitable and connected to the society which it will serve in the future. As a result, it is understood how research universities not only consider relevant teaching for degrees, but also when it comes to Masters and Doctorates, with these latter two disciplines allowing them to achieve a greater level of specialization. This enables them to connect more easily with today’s increasingly technological and global society.

### Pioneering research

Research should not be seen simply as another activity when teaching allows it, “rather as an intellectual motor which inspires teachers, means they are in state of creative tension, and allows them to transmit this knowledge in a creative and useful way for their students and for the society which they hope to improve” [20]. Pioneering research and feedback on this from society, who evaluates, makes use of and requests it, generally represents a key factor which is able to position the institution at a local, national and international level [11].

Research represents these universities’ driving force, and as a result there are important strategies focused on achieving this objective. Research is channeled through links between industry and the university, with the general aim of researching topics which can generate income. An important task is to define a suitable balance, in order to avoid a decrease in the quality of research whilst achieving financial stability.

### Relevance and links with society

“Relevance in society and its links to the university is a key component for these research studies. On one hand these links create substantial incomes which allow the institutions to operate, and on the other hand they keep these universities involved in social needs, at the forefront of progress, research and innovation. Without a doubt, this component is at the core of any institution which aspires to be recognized as a “research university”; as without ideas there are no projects, without projects there are no links to society or quality research which drives current and relevant teaching” [20].

The significant relationship which exists between the three characteristics described is already clear in this final one: relevance and links with society. The answer to why a university is relevant is clear: a university which researches topics requested by the society they serve, a university which as a result of its relevant teaching develops professionals dedicated to meeting social needs, is a relevant and well-connected university. When it comes to reviewing the literature, we can see how these research universities should be linked to local, regional, national and international civil society through companies, institutions, administrations, etc. and the projects required by these [21–22]. Therefore, the research university should be an institution with a social nature (closely linked to society’s needs) with one of its key objectives being the creation of useful knowledge for society [23] [24].

These three previously mentioned characteristics of research universities are present, in an implicit manner, in the main university rankings [25]. It should be noted that, according to various authors [15][26], these university rankings which have become more prominent in the last decade, have arisen as a result of the widespread growth of higher education, competition between universities and the commercialization of tertiary education at a global level. The nature of these rankings means that they are an evaluation tool that should be taken into account and whose results should not be considered as an objective in itself [25].

International rankings such as Shanghai, The Times or Webometrics consider these aforementioned characteristics (albeit with greater or lesser percentage weighting and in a more or less biased manner) within their indicators (Table 1.) As a result, if a university develops these characteristics, this development can lead to an improved position in the rankings [25].

**Table 1:** Link between international rankings and research university characteristics. Created based on [25] [27–29].

	Ranking		
Research University Characteristics	Webometrics	Shanghai	The Times
Relevant teaching	10,00%	13,33%	15,00%
Pioneering research	40,00%	43,33%	42,00%
Relevance and links with society	50,00%	43,33%	43,00%
**Number of institution ranked**	26.000	500	981

## Peruvian context: Law 30220 and SUNEDU

Three main university reforms have been implemented in Peru prior to Law 30220. The first reform, referred to as “autonomy and co-governance”, was established with the Córdoba movement in Argentina, in 1918. During this time, academic quality was not a priority because “mainly small sectors of society with high levels of cultural capital in their household” were enrolled in education, because there were few teachers and “they had high levels of training, often obtained outside of the region”, and in particular, because in this era knowledge was updated slowly.

Following this came a second reform, termed broadly as “commercialization and differentiation”. This was characterized for establishing a model which differentiated public universities from private ones, providing the latter with promotion policies. During this phase, quality standards were not deemed necessary. As a result, it was presumed that students were the ones who could best select their higher education options. In summary, universities started to be considered as clients. A consequence of this reform, and due to widening the offer, new sectors of the population with relatively low cultural capital were able to access higher education, in what can be described as a democratization of professional studies. This led to increased differentiation amongst universities based on the quality of their teaching.

Finally, the third reform of “opening access and internationalization” led to a clear decline in academic quality as a result of reduced public funding per head and the creation of new universities offered to the market without internal or external systems which guarantee the quality of teaching. The number of these new universities was determined by economic interests, which led the State to reduce its investment in public universities [30]. The State created the National Board for Authorizing University Operations (CONAFU) in 1995. This was an entity which controlled the authorization of new universities in the country, which disappeared after a short period of time. In 1996, Law N° 882 was published, known as the “Law for Promoting Investment in Education”, which increased the options for funding private universities. Therefore, by “modernizing the education system and broadening the offer and coverage” as mentioned in the decree, it was demonstrated that access (and not so much quality or knowledge) was the main factor behind the university system’s purpose in Peru [31]. During this last phase, a focus on higher education quality arose, a trend which started in England at the start of the 1990s.

These reforms, and in particular the disproportionate opening of private universities (in the last 25 years more than 75 of these institutions have been set up [32], led to a profound university crisis in Peru. Instead of creating effective mechanisms for the academic management of universities that were already established in the region in order to strengthen them and turn them into world-class research universities, public universities were weakened. Unnecessary competition was created, especially between private universities, under the pretext of educational democratization which would reach the maximum number of people [33]. In addition, Peruvian universities received very modest classifications in the international rankings [27].

This crisis can be understood by looking at the three fundamental shortcomings in the university system which later become the three key action points of the new Law N° 30220. Firstly, the State was not responsible for the administration of education policies at all levels, in an attempt to establish alignment amongst all of these. A national educational project was necessary, not only to provide clear guidance in terms of the direction sought, but also to propose mechanisms which articulate (based on needs) the education system itself [34]. This focus coincides with the World Bank’s recommendations with regards to modelling and improving conventional and traditional universities to align them to the specific demands and needs of the country [4]. Secondly, there was no National System responsible for ensuring teaching quality, so that families and students had guarantees in terms of the education service offered by educational institutions. Thirdly, there was a lack of reaffirmation of the university as a space for building knowledge based on research and integrated training, a new vision which is in keeping with the strategies for establishing world-class research universities [4] [20].

This analysis was used to justify the need to create a new university law: Law 30220. After two years of being debated, Law N° 30220 was finally approved on the 9th July 2014. In order to implement this law, the National Authority for University Education (SUNEDU) was created as an autonomous body forming part of the Education Ministry. This body introduced the mandatory and renewable system for awarding degrees in universities, leading to the disappearance of the authorization of provisional operations which existed under the previous legal framework and had caused the proliferation of private universities. The governing body (SUNEDU) established eight basic conditions for defining the quality of higher education which were chosen by studying and analyzing the systems which were implemented in a number of countries such as Chile, United States, Spain and Ecuador [35–36]. These conditions are as follows:

Condition I. Existence of academic objectives, degrees and qualifications, and study plans. There is a move away from the premise that a university has been able to establish all of this based on an evaluation of the situation and the expectations of future students. This also recognizes the way in which the administrative system which is responsible for its management is formed.

Condition II. Educational offering compatible with the objectives established during planning. For SUNEDU, this condition involves the university being able to show how it creates the budget for its activities and sustains its investment in following years.

Condition III. Adequate infrastructure and equipment to efficiently run classrooms. Everything that is required in a university is included in terms of minimum requirements for security, capacity available and technological equipment in order to guarantee the academic objectives stated in the first condition. This section requires that the facilities for university students should be differentiated from those for basic education, something which is currently a problem in Peru: there are records of universities which operate from offices, old schools or even shopping malls, for example.

Condition IV. Research and development lines. The supervisory entity states that all universities should design research activities led by their own teachers and students, in order to facilitate innovation and knowledge transfer.

Condition V. Availability of qualified teaching staff with no less than 25% of these being full time. With this point, SUNEDU is aiming to achieve greater interaction between students and teachers, including at class level in order to facilitate their learning and create research criteria.

Condition VI. Existence of basic complementary services. The guideline refers to all those medical, social, psychopedagogy and sporting services, among others; which without necessarily being educational, enable them to provide the student with conditions that foster learning and professional development.

Condition VII. Existence of mechanisms for mediation and entry into the job market. In a similar way to the point above, these services (such as job listings or professional placements) are seen as a way of facilitating and complementing students’ development, especially those who require experience in the work place.

Condition VIII. Transparency. SUNEDU requires that all universities make information on their academic offering public, as well as their results and the quality of services they offer, with the aim of helping students make better decisions and introducing a competitive aspect to the education and professional sector.

Through these conditions, the SUNEDU also aims to use this law to improve the position of Peruvian universities within the international rankings, which are led by research universities in which the characteristics intrinsic to these universities prevail. As a result, if the SUNEDU’s law aims to improve the ranking of Peruvian universities, it is important that the stated conditions are aligned to the main characteristics of research universities.

## Methodology

In order to assess the conditions imposed by SUNEDU in terms of the aforementioned characteristics which research universities should possess, a Delphi panel was chosen due to the lack of impartial information which was liable to be substituted for expert judgment [37–38]. Furthermore, this methodology is positive in the sense that it reduces the negative effects which face-to-face interviews could have had, although its main downside is that it is subject to the biases of those experts involved in the panel [39].

Based on the large number of experts participating in successive conferences on research universities, a number of these were selected which in order to form part of the panel should possess the following characteristics: experience managing national and international research projects, experience in administrative roles in universities (chancellor or vice-chancellor), accredited teaching experience (at least 10 years) and should have carried out relevant research at an international level. This group of experts was formed of 10 members of the international academic community, belonging to different universities across Europe, Latin America and North America in the following countries: United States, Spain, Peru, Ecuador, Chile and Argentina. These countries were chosen as their university systems had been studied by SUNEDU during the process of selecting the established conditions. In addition, it was ensured that these experts had some kind of link to Peruvian Universities through agreements or research projects. The size of the group was considered suitable as it fell within the optimum size of between 7 and 30 experts recommended by [40].

As a first step in the methodology, an open question was asked regarding the research university’s characteristics and the conditions imposed by SUNEDU. This first stage was carried out in-person during the General University Conference which took place in La Plata (Argentina) and the experts’ contributions were gathered in order to create the questionnaires.

The second stage involves setting up the questionnaires and sending these to each of the experts. The questionnaires were designed so that the experts could score the basic conditions required to define the quality of SUNEDU’s higher education teaching and its relationship with the characteristics of the research university. A scoring scale of 1 to 9 was proposed, in which 1 indicates very low correlation, 3 low correlation, 5 average correlation, 7 high correlation, and 9 very high correlation.

Consensus was reached after three rounds of consultation. The Delphi panel was completed in a third stage in which the responses sent by the experts were included and scoring was carried out.

During the panel, consensus was sought amongst the experts. This was scored using an interquartile range, defined as the interquartile range (k) between the 3rd quartile (q3) and the first (q1), so “k” = q3-q1 with variations of less than 20% being acceptable. The stability of the responses following the consensus was calculated using the relative interquartile range (RIR) of the two rounds carried out, with stability being obtained when the results of the RIR are within the +-0.2 value range [39].

## Results

The results of the panel show a consistent directional trend, because as previously stated, the basic conditions imposed by SUNEDU are integral to each of the research university’s characteristics. The results and consensus indexes obtained through the Delphi panel throughout the three rounds are shown in Table 2 (results relating to teaching), Table 3 (results relating to research), Table 4 (results relating to relevance and links with society) and in S1 Dataset.

**Table 2:** Results of the Sunedu-Teaching conditions.

	INTRSINSIC CHARACTERISTICS OF RESEARCH UNIVERSITIES																	
	TEACHING																	
	Round 1 (1R)						Round 2 (2R)						Round 3 (3R)					
Sunedu Conditions ConditConditions	1	3	5	7	9	IC	1	3	5	7	9	IC	1	3	5	7	9	IC
**Condition I**	0%	20%	10%	10%	60%	4,5	0%	10%	0%	0%	90%	0,0	0%	0%	0%	0%	*100%	0,0
**Condition II**	0%	20%	20%	20%	40%	4,5	0%	10%	0%	0%	90%	0,0	0%	0%	0%	0%	*100%	0,0
**Condition III**	0%	20%	0%	30%	50%	3,0	0%	0%	0%	20%	80%	2,0	0%	0%	0%	0%	*100%	0,0
**Condition IV**	40%	0%	20%	30%	10%	6,0	50%	10%	0%	30%	10%	6,0	70%	20%	10%	0%	0%	2,0
**Condition V**	10%	0%	0%	60%	30%	2,0	0%	0%	0%	60%	40%	2,0	0%	0%	0%	*60%	40%	2,0
**Condition VI**	20%	0%	20%	20%	40%	5,0	0%	0%	10%	10%	80%	0,5	0%	0%	0%	0%	*100%	0,0
**Condition VII**	40%	0%	10%	30%	10%	6,0	80%	0%	0%	20%	0%	0,5	100%	0%	0%	0%	0%	0,0
**Condition VIII**	0%	0%	10%	70%	20%	0,5	0%	0%	10%	80%	10%	0,0	0%	0%	10%	*80%	10%	0,0

*“High” and “very high” correlation (7 or 9) in Round 3.

**Table 3:** Results of the Sunedu-Research conditions.

	INTRSINSIC CHARACTERISTICS OF RESEARCH UNIVERSITIES																	
	RESEARCH																	
	1R						2R						3R					
Sunedu Conditions	1	3	5	7	9	IC	1	3	5	7	9	IC	1	3	5	7	9	IC
**Condition I**	50%	10%	0%	30%	10%	6,0	80%	10%	0%	10%	0%	0,5	100%	0%	0%	0%	0%	0,0
**Condition II**	40%	20%	10%	20%	10%	6,0	70%	10%	10%	10%	0%	2,5	100%	0%	0%	0%	0%	0,0
**Condition III**	40%	20%	10%	10%	20%	6,5	70%	10%	10%	0%	10%	2,5	100%	0%	0%	0%	0%	0,0
**Condition IV**	0%	0%	0%	40%	60%	2,0	0%	0%	10%	10%	80%	0,5	0%	0%	0%	0%	*100%	0,0
**Condition V**	0%	20%	0%	40%	40%	3,0	0%	0%	0%	40%	60%	2,0	0%	0%	0%	40%	*60%	2,0
**Condition VI**	50%	10%	40%	0%	0%	4,0	80%	0%	20%	0%	0%	1,0	100%	0%	0%	0%	0%	0,0
**Condition VII**	50%	0%	20%	20%	10%	6,0	90%	0%	0%	10%	0%	0,0	100%	0%	0%	0%	0%	0,0
**Condition VIII**	0%	10%	10%	70%	10%	2,0	0%	10%	0%	70%	20%	0,5	0%	0%	0%	*80%	20%	0,5

*“High” and “very high” correlation (7 or 9) in Round 3.

**Table 4:** Results of the Sunedu-Relevance & links with society conditions.

	INTRSINSIC CHARACTERISTICS OF RESEARCH UNIVERSITIES																	
	RELEVANCE AND LINKS WITH SOCIETY																	
	1R						2R						3R					
Sunedu Conditions	1	3	5	7	9	IC	1	3	5	7	9	IC	1	3	5	7	9	IC
**Condition I**	60%	0%	0%	30%	10%	6,0	60%	10%	10%	20%	0%	4,5	100%	0%	0%	0%	0%	0,0
**Condition II**	40%	20%	10%	10%	20%	6,5	60%	10%	10%	20%	0%	4,5	100%	0%	0%	0%	0%	0,0
**Condition III**	40%	20%	10%	20%	10%	6,0	80%	0%	20%	0%	0%	1,0	100%	0%	0%	0%	0%	0,0
**Condition IV**	40%	0%	0%	30%	30%	8,0	70%	0%	0%	10%	20%	6,5	100%	0%	0%	0%	0%	0,0
**Condition V**	40%	30%	0%	20%	10%	6,0	60%	10%	10%	20%	0%	4,5	100%	0%	0%	0%	0%	0,0
**Condition VI**	50%	10%	30%	0%	10%	4,0	80%	0%	20%	0%	0%	1,0	100%	0%	0%	0%	0%	0,0
**Condition VII**	10%	0%	0%	70%	20%	0,5	10%	0%	0%	0%	90%	0,0	0%	0%	0%	0%	*100%	0,0
**Condition VIII**	0%	0%	0%	70%	30%	2,0	10%	0%	0%	70%	20%	0,5	0%	0%	0%	*80%	20%	0,5

*“High” and “very high” correlation in Round 3 (7 or 9).

In the table above, the “high” and “very high” (7 or 9) responses have been marked with an asterisk, which shows that the consensus is statistically significant. This high level of consensus means that possible biases can be ruled out which are derived from the experts’ different origins, especially amongst those belonging to Peruvian universities.

If we look at the stability of the responses, Table 4 shows the index of variation of the relative interquartile range (RIR) from the various panel rounds. We can see from Table 5 that there is a variation between the first and second rounds as well as the second and third rounds. The variation in the interquartile range is considered acceptable when it is within an interval of (-0.2, 0.2). Within the asterisks “*”, those responses which are considered stable can be seen.

**Table 5:** Variation of the interquartile range between different rounds.

	Intrinsic Characteristics of Research Universities					
	Teaching		Research		Rel. & link with society	
Sunedu Conditions	RIR R1-R2	RIR R2-R3	RIR R1-R2	RIR R2-R3	RIR R1-R2	RIR R2-R3
**Condition I**	0,63	0,00	1,30	0,28	0,06	1,61
**Condition II**	0,68	0,00	0,44	1,14	0,02	1,61
**Condition III**	0,36	0,06	0,44	1,04	1,02	0,56
**Condition IV**	-0,30	0,56	0,20	0,06	-0,49	2,03
**Condition V**	0,03	0,00	0,17	0,00	0,06	1,61
**Condition VI**	0,75	0,06	0,87	0,56	0,69	0,56
**Condition VII**	1,19	0,31	1,58	0,00	0,07	0,00
**Condition VIII**	0,07	0,00	0,19	0,01	0,19	0,01

Based on this data, the objective is to observe which characteristics the different SUNEDU conditions have an impact on. In Table 6, which is shown below, an “X” indicates the characteristics of the research university which the basic conditions have a high or very high impact on, whilst also having an acceptable level of response stability (RIR).

**Table 6:** Conditions imposed by SUNEDU in which the relationship with the characteristics of the research university is high or very high. The RIR of the responses is shown in brackets.

	Teaching	Research	Link with society
**Condition I**	X (0,00)		
**Condition II**	X (0,00)		
**Condition III**	X (0,06)		
**Condition IV**		X (0,06)	
**Condition V**	X (0,00)	X (0,00)	
**Condition VI**	X (0,06)		
**Condition VII**			X (0,00)
**Condition VIII**	X (0,00)	X (0,01)	X (0,01)

## Discussion and conclusions

As shown in the Delphi panel results, the basic conditions established by SUNEDU cover all of the characteristics of a research university. The consensus and the stability of the responses when the extent of the relationship is high or very high invalidate the existing relationship. However, the research is limited to the chosen experts and the established membership conditions. In terms of the results, it is worth highlighting that “teaching” is the characteristic which is most frequently repeated in the various conditions. This situation could have arisen as a response by SUNEDU to the problem with teaching quality which Peru has faced in recent years [36]. The conditions presented by SUNEDU therefore have a clear teaching component, although the results also show characteristics related to research, links with society as well as conditions which cover all of the characteristics (such as VIII, transparency).

It should be noted that three of the basic conditions (IV, V and VI) cover aspects relating to research, a characteristic which we could argue is currently lacking in Peruvian universities, which are facing the challenge of training researchers in international research universities and trying to obtain the required resources which allow them to “support” investment in research. In line with these conditions and the protection under law 30220, Peruvian universities (almost as a whole) have established bodies dedicated to research governance, through vice-chancellors or directors. This is in order to facilitate and promote the creation of new strategic areas of research as well as the possibility of developing these.

The results also show that the links with society are considered (as stated in the SUNEDU’s document) from the point of view of assessment and entry into the job market, something which is probably driven by the strong growth in the country [41] in recent years and the need for a specialized workforce to enable this growth to be sustainable. These links with society should be univocal relationships (from the university to society) and not biunivocal as suggested by the characteristics of the research university [20]. This could be due to the characteristics of the Peruvian economy, which is mainly based on exploiting raw materials and therefore does not necessarily require a strong scientific or technological basis for the country’s development.

If we look at the results we can see that the “relevance and links to society” characteristic has had a high level of instability linked to low or very low correlation scores with some of the conditions imposed by SUNEDU. This could be due to the high interrelation and dependency which exists between the research university’s intrinsic characteristics and the others. During the panel, the experts’ comments reject this aspect, indicating that the link between conditions I, II, IV, V & VI imposed by SUNEDU and this characteristic have a secondary and indirect link, which could be high if it arises as a result of complying with the conditions imposed.

The fact that the SUNEDU’s conditions cover, to a greater or lesser extent, the characteristics of the research university means that Law 30220 is laying the foundations for the transformation of Peruvian research universities. Therefore, in the future, its implementation and validity can facilitate the improvement of Peruvian universities in the international rankings. As previously discussed, these apply indicators linked to the characteristics of research universities. It is important to mention that the results obtained in these rankings should not be interpreted as a goal in itself, but rather as an external evaluation of these universities’ evolution and development.

## Supporting information

S1 Dataset Delphi Panel data. Data of the 3 rounds in the Delphi Panel and analysis of the data.
(XLSX)
